# What it takes to become a physician scientist in a low- and middle-income country

**DOI:** 10.1371/journal.pgph.0004234

**Published:** 2025-04-23

**Authors:** Beatriz Barreto-Duarte, Simon C. Mendelsohn, Elsa Du Bruyn, Bruno B. Andrade

**Affiliations:** 1 Programa Pós-graduação de Clínica Médica. Faculdade de Medicina, Universidade Federal do Rio de Janeiro, Rio de Janeiro, Brazil; 2 Institute of Research in Priority Populations, Instituto Monster de Ensino, Assistência, Pesquisa e Desenvolvimento Tecnológico em Saúde, Salvador, Brazil; 3 Multinational Organization Network Sponsoring Translational and Epidemiological Research (MONSTER) Initiative, Salvador, Brazil; 4 Instituto de Pesquisa Clínica e Translacional (IPCT), Faculdade ZARNS, Clariens Educação, Salvador, Brazil; 5 Laboratório de Pesquisa Clínica e Translacional, Instituto Gonçalo Moniz, Fundação Oswaldo Cruz, Salvador, Brazil; 6 South African Tuberculosis Vaccine Initiative, Institute of Infectious Disease and Molecular Medicine and Division of Immunology, Department of Pathology, University of Cape Town, Cape Town, South Africa; 7 Wellcome Centre for Infectious Disease Research in Africa and Institute of Infectious Disease and Molecular Medicine, University of Cape Town, Observatory, Cape Town, South Africa; 8 The BRIGHT lab, Instituto Monster de Ensino, Assistência, Pesquisa e Desenvolvimento Tecnológico em Saúde, Salvador, Brazil; University of Washington Department of Global Health, UNITED STATES OF AMERICA

## Abstract

Physician-scientists, who have dual medical and advanced research training, are a scarce and valuable asset. They bridge clinical practice and research, address critical medical challenges with a scientific perspective, and drive innovation by translating discoveries into patient care. Physicians with research expertise are particularly adept at critically evaluating scientific literature to improve their practice and ensure that they provide up-to-date, individualised, and evidence-based care to their patients. However, the path to becoming a physician-scientist in Low- and Middle-Income Countries (LMICs) is fraught with challenges. In this article, we explore the difficulties faced by physician-scientists in LMICs, including lengthy and arduous training, systems that favour eminence-based over evidence-based medicine, and financial disincentives for pursuing a dual career in medicine and research. The article also highlights the significant underrepresentation of women in medical and scientific fields, compounded by gender-specific challenges such as balancing motherhood with career demands, gender pay gaps, and the lack of supportive and affirmative policies. We advocate for reforms in medical education to create a more supportive environment for aspiring physician-scientists. Addressing these issues can help LMICs enhance the contribution of physician-scientists to global health and scientific advancement.

## Introduction

On the first day of medical school, professors often ask, “Why did you choose to study medicine?” Many doe-eyed students state that they see it as their “calling” or vocation, while others cite altruistic aspirations. In many cultures, medical doctors are highly esteemed in their communities, which further motivates prospective graduates. Medical students, particularly in Low- and Middle-Income Countries (LMICs), rarely consider pursuing a career as physician-scientists, despite the diversity and unique challenges within these regions. That elucidate why the health systems in LMICs struggle with outdated practices due to insufficient research capacity, as seen in the limited availability of innovative treatments for endemic diseases. Furthermore, most medical curricula in LMICs neither inspire nor support medical students in pursuing a career as a physician-scientist. This represents a critical missed opportunity, as LMICs often face high burdens of both communicable and non-communicable diseases. Health sector policymakers and the medical fraternity in these countries would be hard pressed to find a better cadre of professionals to grapple with these problems than physician-scientists. Their dual skill set allows them to prioritize clinically relevant research questions based on observed realities of human health and disease, potentially leading to a greater impact. However, training, supporting, and retaining these highly skilled individuals who bridge the divide between clinical medicine and basic science requires significant investment. In this article, we outline the manifold challenges that physician-scientists face in LMICs and explore potential opportunities to overcome them.

### What defines a physician-scientist?

A physician-scientist is a doctor who has completed formal research training (PhD or equivalent) beyond their conventional medical education and dedicates part of their career to scientific endeavours. In LMICs, physicians proficient in basic science are a rare commodity [1,2]; fewer still hold both a medical degree and a PhD. Physician-scientists in LMICs are typically affiliated with the health science faculties of universities, where they balance clinical, academic, and teaching responsibilities while conducting research. It is almost unheard of for a physician-scientist to be able to dedicate themselves entirely to scientific research.

### Tortuous educational pathways for physicians in LMICs

The expected pathway for medical graduates typically involves transitioning into clinical residency, a phase that demands grueling hours and prioritizes specialization over a broader understanding of research or systemic health issues. This singular focus on clinical duties leaves little room for the integration of research methodologies or the application of evidence-based practices. Furthermore, reliance on observational learning, rather than structured teaching of scientific principles, contributes to significant gaps in knowledge [3]. Trainees are often exposed to outdated practices passed down through repetition, with limited engagement in discussions about critical appraisal of research or its application to clinical care.

Medical training in LMICs often exposes students to healthcare systems deeply impacted by vulnerability and poverty, where the health conditions of patients are intrinsically linked to social determinants such as housing, income, and education. While LMICs are not homogeneous, exhibiting marked social, cultural, and educational differences, it is nonetheless expected that most share common challenges related to the burden of poverty and inequity, which shape both the training environment and the healthcare systems themselves. This constant interaction with resource-constrained settings and underserved populations amplifies stress levels among medical students, as they are frequently placed in situations where their ability to provide care is limited by factors outside their control [4]. Witnessing these inequities on a daily basis often leads to a heightened sense of helplessness, further exacerbating mental health challenges like stress, anxiety, and burnout.

This environment reveals a paradox: while students are trained to improve patient health through scientific advancements and clinical skills, they often find themselves unable to apply this knowledge in practice [5]. The lack of resources, financial constraints of patients, or unavailability of appropriate therapies [6] frequently undermines their efforts, creating frustration and reinforcing a sense of inadequacy.

### Deficiencies in medical curriculum and research training in LMICs

Advances in medicine over the last century have taken place against the backdrop of technological innovations that have enabled rapid scientific progress and fostered communication between physicians and scientists. Despite this, a critical shortcoming in medical curricula across LMICs is the lack of integration between research and clinical practice, which limits the development of evidence-based care [3]. This disconnect fosters an environment where clinical decisions often rely on tradition or anecdotal evidence, rather than scientifically validated approaches [7]. The COVID-19 pandemic underscored this deficiency, as pseudoscientific practices gained traction even among healthcare providers, with some physicians touting treatments that have not been proven beneficial through randomised controlled trials. This issue is particularly prevalent in LMICs, like South Africa [8] and Brazil [9]. For physicians, this problem is not driven by desperation, but by a lack of understanding of the principles of evidence-based medicine. Now more than ever, there is a pressing need to critically re-evaluate medical curricula to address this deficiency.

This gap is further exacerbated by the absence of structured opportunities for students to engage with research methodologies or evidence-based frameworks during their education. Medical training in LMICs continues to emphasize practical repetition over analytical understanding, leaving students ill-equipped to evaluate or implement findings from scientific literature. Many physicians find basic science and biostatistics intimidating [10,11], but this need not be the case. Often the problem is the introduced without context or clear application to clinical care, which discourages students from pursuing deeper engagement with research. Even the understanding of the basic principles can help bridge the gap between bench and bedside when facilitated by a physician-scientist. Unfortunately, trainees in LMICs often have limited exposure to physician-scientists [12]. While they may attend a few lectures by professors with PhDs, these lectures are likely to focus predominantly on clinical content. The process of translating a clinical question into a testable hypothesis, whether through a trial or experiment, is best taught by an experienced physician-scientist with extensive expertise. This type of education is lacking in LMICs, partly because the medical fraternity does not fully recognise the value of physician-scientists, contributing to their scarcity in these regions [12].

Furthermore, very few intercalated MD-PhD training programs exist in LMICs, despite their success and prestige in high-income countries (HICs) [13].These programs allow medical students to pause their medical studies to pursue research for a set period, with the ultimate goal of obtaining a research degree (Masters or PhD) and then returning to the regular medical curriculum [14].This approach offers the further advantage of reducing the overall time needed to obtain both degrees. Although expanding such programs in LMICs would be highly beneficial, it would require substantial funding for both research costs and student stipends. This is particularly challenging in resource-constrained settings, where the healthcare system relies heavily on young physicians to meet the majority of public healthcare needs, and there is limited governmental financial capacity to support the costly training of physician-scientists.

### The diminishing returns of becoming a physician-scientist in LMICs

Pursuing a career as a physician-scientist in LMICs is fraught with challenges. Many physicians fascinated by science end up pursuing it as a side hustle, which diminishes productivity and significantly reduces the chances of securing research funding [3]. A major discouraging factor is the time required to meet the degree requirements for becoming a full-fledged physician-scientist. This can result in significant student debt and financial pressure, especially without scholarships or fellowships. Additional time is required if specialization or residency is pursued, leading to a heavy clinical workload. Moreover, some LMICs require up to three years of obligatory clinical work in the public health sector before a physician can register as an independent practitioner. While a clinician, once registered, gains the ability to set up a private practice and generate income directly from patient consultations, researchers face an entirely different reality. A career in research does not offer equivalent financial autonomy or the flexibility to operate independently, as the majority of research funding is tied to institutions or grants [15]. This disparity often makes clinical practice more appealing to young professionals, as it provides a clearer and more immediate pathway to financial stability and career progression

Transitioning from clinical practice to full-time research often results in lower earnings, and post-PhD, there is usually no salary increase unless it leads to a professorship or career advancement in academia [2]. These factors, along with better opportunities for research funding and career development abroad, often lead to physician-scientists leaving LMICs for HICs – a phenomenon known as the “brain-drain [16–21]”.

Paradoxically, it is estimated that around 30% of all healthcare resources are wasted, with unnecessary medical tests and procedures accounting for about 10% of healthcare costs [7,22,23]. This inefficiency is particularly detrimental in low- and middle-income settings, where resources are limited. The presence of physician-scientists plays a crucial role in advancing medicine by ensuring that high-level evidence-based practices are developed and applied effectively. Their expertise contributes not only to the development of new treatments and medications but also to optimizing the use of diagnostic tests and procedures, reducing unnecessary interventions. By fostering an environment where research and clinical practice coexist, physician-scientists can significantly enhance access to high-quality care and improve the cost-effectiveness of healthcare systems [7], ultimately benefiting patients and ensuring that limited resources are used more efficiently.

### Impact of political and instability and polarization

Political instability further complicates the pathway for physician-scientists by creating an unpredictable research environment. In countries with frequent government changes or polarized political landscapes, such as Latin America [24–26], research priorities and funding allocations often shift dramatically, disrupting long-term scientific projects [27]. For physician-scientists, this instability makes it difficult to plan a career path in research or to rely on sustained funding for projects that require years of dedication. Projects launched with government backing may face delays, funding cuts, or cancellations if political priorities shift, stalling progress and discouraging professionals from committing to a research-focused career.

Additionally, political polarization can undermine public trust in science, as seen in various LMICs during the COVID-19 pandemic [25,28,29]. When health interventions become politicized [30], community compliance decreases [31], and physician-scientists encounter resistance to evidence-based practices [30]. This opposition hampers immediate health efforts and demoralizes the scientific community, making it challenging to inspire and retain the next generation of physician-scientists.

### Structural, ethical, and logistical challenges

In LMICs, the pursuit and maintenance of a career as a physician-scientist are significantly hindered by structural, ethical, and logistical barriers that obstruct individual career paths and limit broader scientific and public health advancements. These challenges are intertwined with deeper socioeconomic and infrastructural limitations that create an arduous environment for medical professionals seeking to engage in clinical and translational research.

### Structural barriers

A primary structural barrier in LMICs is the lack of dedicated financial support for medical research and scientific training programs [32]. Unlike high-income countries, where research infrastructure is often well-established, LMICs frequently lack the facilities, equipment, and financial resources necessary to support research activities [33]. Additionally, the healthcare systems in LMICs are often underfunded and overburdened, exacerbating these issues [34]. Many patients assisted in these settings face significant socioeconomic challenges, including poverty, limited access to basic healthcare services, and financial constraints that hinder their ability to participate in or benefit from research [6]. Young physician-scientists face restrictions in accessing modern laboratories, specialized technologies, and research funding, limiting their ability to gain hands-on research experience or develop in-depth expertise in critical areas. Consequently, physician-scientists in these settings are often left without the resources they need to conduct high-quality research on issues that disproportionately affect LMIC populations, such as infectious diseases, surgical conditions and chronic health conditions.

### Ethical challenges

Ethical and regulatory systems in LMICs also present significant obstacles for physician-scientists. While ethical oversight is crucial for maintaining research integrity, the ethical review processes in many LMICs are underdeveloped or inconsistently applied, creating lengthy approval times and administrative hurdles [33]. This is especially problematic for young researchers who require timely project approvals to advance their careers and contribute meaningfully to public health research. The lack of a streamlined, efficient process can delay research projects indefinitely, often discouraging early-career scientists from pursuing independent research in their home countries.

Furthermore, navigating complex ethical landscapes in LMICs involves addressing cultural and linguistic differences that complicate the informed consent process and participant recruitment [32], especially in diverse and underserved populations. Physician-scientists may face ethical dilemmas as they attempt to balance the need for scientific rigor with respect for local values and beliefs [35]. The scarcity of well-trained ethics review board members and the limited resources available to support them further exacerbate these issues, leading to delays and potential inconsistencies in ethical evaluations [36]. These challenges can deter early-career physician-scientists from initiating projects and contribute to a general lack of research output from LMICs, further marginalizing these regions in the global research landscape.

### Logistical hurdles

Logistical barriers, such as unreliable access to research data, inconsistent supply chains, and limited laboratory capacity, present additional challenges that impact both the quality and sustainability of research. In LMICs, physician-scientists often work with limited or outdated resources, which can affect the accuracy and reliability of their findings. The absence of robust data collection systems and data-sharing infrastructure limits the ability to conduct longitudinal studies or large-scale clinical trials that are essential for evidence-based healthcare [33]. Additionally, logistical challenges in recruiting and retaining participants from rural or underserved communities further hinder research that could inform effective health interventions [32].

International collaborations have been effective in mitigating some of these logistical challenges. For instance, partnerships with organizations like the World Health Organization and regional health bodies have provided LMIC researchers with access to global resources, training, and funding [34], enabling them to address local health challenges more effectively [28,29]. Leveraging digital tools and remote technologies can further support data collection and participant engagement in geographically challenging settings.

### Gender inequality among LMIC physician-scientists

There is a significant gap in the representation of women in leadership roles within the medical field and scientific research [37], which becomes even more pronounced in LMICs. While women constitute up to 70% of the health and care workforce globally, they remain significantly underrepresented in senior positions, holding less than 20% of leadership roles in academic or research settings [37]. This disparity is especially pronounced in scientific fields, impacting career progression and citation rates. Several studies have shown that women are largely underrepresented in medical research [37–39]. Although female physicians do not yet make up the majority of the medical workforce, the feminization of the profession is underway, with women being overrepresented in younger age groups in various regions [37,40,41].

The challenges of balancing childbearing with a demanding career as a physician-scientist are significant [42]. However, progress in academic medicine continues remains slow, with women accounting for less than 30% of clinical faculty [40]. Understanding how this underrepresentation affects high-impact research is crucial, as it introduces implicit bias into the research agenda, which can influence future clinical practice. For instance, the underrepresentation of women in clinical research can result in gender-blind research agendas that overlook critical health issues predominantly affecting women. A lack of supportive policies, such as maternity leave and flexible working hours, exacerbates the struggle for work-life balance [43]. In addition, women in LMICs face a notable gender pay gap, with female physicians earning less than their male counterparts, which further discourages women from pursuing or continuing careers in medical research [39,43–45]. Programs that address the gender pay gap, such as targeted salary adjustments and transparency in academic institutions, have been successful in reducing disparities in some LMICs, but these remain rare [45].Moreover, there is a shortage of affirmative policies designed to support and promote women in the medical and scientific fields [38,44].. Examples of effective initiatives include the Women in Global Health Leadership Fellowship, which supports African women in health systems leadership [46], and the Mwele Malecela Mentorship Program [47], focused on advancing female leadership in combating neglected tropical diseases in Africa. Similarly, India has implemented the WomenLift Health Leadership Journey, [48] a government-supported program aimed at empowering women leaders in health research.

Governments in several LMICs have also introduced awards and funding initiatives specifically for women in science and medicine. For instance, the Indian Department of Science & Technology offers the ‘Women Scientists Scheme’ to encourage women to pursue research careers [41]In Brazil, the ‘Programa Mulheres na Ciência’ provides financial incentives and recognition for female researchers leading innovative projects [42]. These awards provide financial support and visibility, inspiring younger generations to enter and thrive in scientific fields. However, such efforts remain insufficient to bridge the significant gap in gender representation and to address persistent disparities in salaries and career incentives faced by women in these regions. The absence of mentorship programs and networks that could nurture female leadership contributes to the underrepresentation of women in senior and decision-making roles. It is crucial to have more women in leadership roles to advocate for and implement policies that support the career advancement of other women.

### What it takes to build the path to becoming a physician-scientist

Despite the obstacles, the pathway to become a physician-scientist in LMICs is possible with strategic planning and leveraging available resources. One of the most critical steps is identifying potential mentors and networking opportunities. Conferences and academic events provide an excellent platform to meet experienced professionals and build connections. Aspiring physician-scientists can also initiate their research journey by exploring secondary data projects, such as systematic reviews and meta-analyses, which foster autonomy and require fewer resources.

A crucial aspect of approaching potential mentors is thorough preparation. Before reaching out, it is essential to have a well-thought-out idea that aligns with the mentor’s area of expertise. One effective strategy is to formulate a specific research question suitable for a systematic review and invite the mentor to act as the senior author. This approach establishes a collaborative relationship as well as demonstrates initiative and an understanding of the mentor’s field.

Moreover, aspiring physician-scientists can explore funding opportunities through national and international research consortia, such as research fellowships or career awards offered by organizations like BRICS [43] or Regional Prospective Observational Research for Tuberculosis (RePORT) [44]. Additionally, networks such as the Consortium for Advanced Research Training in Africa (CARTA) [45], the South Asian Clinical Toxicology Research Collaboration (SACTRC) [46], and the Latin American Network for Education in Health Research (LANEHR) [47] offer region-specific programs that foster capacity-building and cross-border collaborations. These initiatives provide platforms for mentorship, funding, and skill development tailored to LMIC contexts.

These funding mechanisms and networks can provide dedicated time and resources for research, enabling individuals to focus on building their expertise and contributing to impactful projects. While the journey to becoming a physician-scientist is undoubtedly challenging, it is immensely rewarding. For the individual, it offers a unique opportunity to merge clinical expertise with scientific inquiry, leading to personal and professional fulfillment. For the healthcare system and the nation, this dual expertise enhances the quality of patient care and drives advancements in science and technology, ultimately contributing to the country’s development and global standing in research and innovation. After establishing a relationship with a mentor and developing foundational scientific knowledge, aspiring physician-scientists may consider pursuing formal training pathways such as an MD-PhD program. Alternatively, they can opt for advanced academic qualifications like a Master’s degree, a PhD, or even post-doctoral training to further solidify their expertise and open doors to academic and research-oriented careers.

### A global perspective and future directions

Countries like the United States and Germany offer well-integrated and heavily supported MD-PhD programs, providing streamlined pathways for aspiring physician-scientists with ample funding, mentorship, and institutional backing. In contrast, LMICs face significant gaps in training and resources, limiting the development of physician-scientists who are essential to addressing local health challenges.

To bridge these gaps, reforms in LMICs must address structural, educational, and cultural barriers. Structurally, dedicated funding mechanisms are needed to establish and sustain research infrastructure, including laboratories, data-sharing systems, and ethical oversight frameworks. These investments should prioritize underserved and rural areas, where disparities in resources are most pronounced, while also promoting gender equity through targeted policies and funding opportunities.

Educational reforms should embed evidence-based medicine, critical thinking, and biostatistics into medical training at all levels. Expanding MD-PhD programs tailored to LMIC contexts, with adequate funding for research and student support, can provide an integrated pathway for training physician-scientists. Mentorship opportunities should be strengthened by fostering interactions with experienced physician-scientists who can guide and inspire trainees.

Cultural shifts are equally vital to fostering a research-oriented environment. Universities and healthcare institutions must prioritize research, incentivize faculty engagement, and promote interdisciplinary collaboration. Physician-scientists should be recognized as key contributors to healthcare innovation, bridging the gap between clinical practice and scientific inquiry. International partnerships can further bolster local capacity and align research priorities with regional needs.

Addressing the shortage of physician-scientists in LMICs would also mitigate the prevalence of “helicopter research,” where external researchers from the Global North set the agenda and often neglect local priorities. By empowering local physician-scientists to lead ethically grounded, community-driven research, LMICs can ensure that studies address their specific health challenges while benefiting their populations, promoting equity.

Reforms in education and infrastructure, coupled with cultural and systemic changes, can position LMICs to build a sustainable pipeline of physician-scientists. These efforts require collaboration among governments, universities, healthcare institutions, and international organizations. By fostering innovation and prioritizing evidence-based practices, LMICs can equip a new generation of physician-scientists to drive healthcare advancements and contribute meaningfully to global health.
